# Reliability of the Dynavision task in virtual reality to explore visuomotor phenotypes

**DOI:** 10.1038/s41598-020-79885-9

**Published:** 2021-01-12

**Authors:** Yvan Pratviel, Veronique Deschodt-Arsac, Florian Larrue, Laurent M. Arsac

**Affiliations:** 1 Univ. Bordeaux, CNRS, Laboratoire IMS, UMR 5218, 33400 Talence, France; 2 CATIE, Centre Aquitain des Technologies de l’Information et Electroniques, Talence, France

**Keywords:** Cognitive neuroscience, Oculomotor system, Rehabilitation

## Abstract

Daily-life behaviors strongly rely on visuomotor integration, a complex sensorimotor process with obvious plasticity. Visual-perceptive and visual-cognitive functions are degraded by neurological disorders and brain damage, but are improved by vision training, e.g. in athletes. Hence, developing tools to evaluate/improve visuomotor abilities has found echo among psychologists, neurophysiologists, clinicians and sport professionals. Here we implemented the Dynavision visuomotor reaction task in virtual reality (VR) to get a flexible tool to place high demands on visual-perceptive and visual-cognitive processes, and explore individual abilities in visuomotor integration. First, we demonstrated high test–retest reliability for the task in VR among healthy physically-active students (n = 64, 32 females). Second, the capture of head movements thanks to the VR-headset sensors provided new and reliable information on individual visual-perceptual strategies, which added significant value to explore visuomotor phenotypes. A factor analysis of mixed data and hierarchical clustering on principal components points to head movements, video-games practice and ball-tracking sports as critical cues to draw visuomotor phenotypes among our participants. We conclude that the visuomotor task in VR is a reliable, flexible and promising tool. Since VR nowadays can serve e.g. to modulate multisensorial integration by creating visual interoceptive-exteroceptive conflicts, or placing specifically designed cognitive demand, much could be learned on complex integrated visuomotor processes through VR experiments. This offers new perspectives for post brain injury risk evaluation, rehabilitation programs and visual-cognitive training.

## Introduction

Most of the daily activities that allow humans to behave adequately in a given environment impose a number of perceptual, cognitive and motor demands. Most of the time, the visual-motor and visual-cognitive functions exhibit sufficient flexibility and robustness to face the daily requirements in an appropriate way, at last in healthy people. Visual perception and eye-hand coordination operate in a very short time thanks to rapid processing in visuomotor integration, an essential sensorimotor system.

It seems that some people show greater visuomotor skills than others^1^, which could be an asset in some situations, e.g. driving a car, piloting an aircraft, or any situation where advanced digital technologies allow returning a huge number of spatially distributed information to human operators.

**Figure 1:**
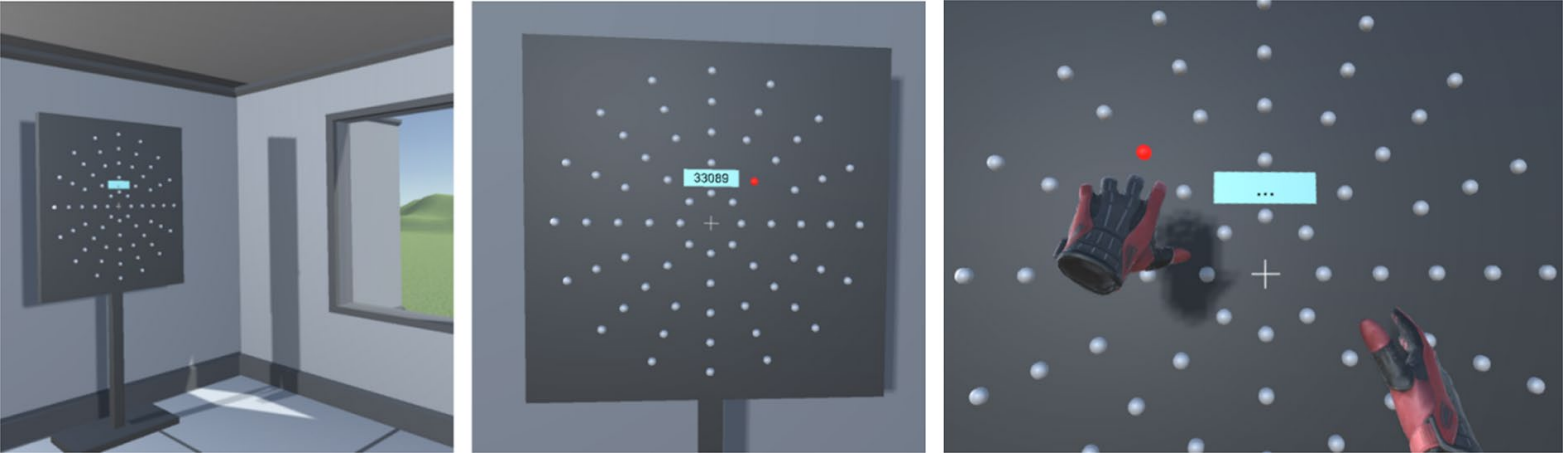
Left panel: large view of the designed virtual environment. Middle panel: cognitive condition of the VMVR task, with numbers appearing on the LCD screen. Right panel: finer view of the display illustrating the virtual buttons arranged in concentric circles with on illuminated target. Gloves represents the controllers the user holds in his hands.

Beyond natural phenotypes, visuomotor integration demonstrates high plasticity. This plasticity is exemplified by degraded functions associated with neurological disease, which strongly contrasts with enhanced eye-hand coordination in sport experts after practice of vision training. In both cases there is an emerging need to place high visual-perceptive and cognitive-motor demands, e.g. in rehabilitation programs to restore impaired visuomotor integration associated to brain damage^2,3^, or in athletic programs to improve psychomotor skills and ultimately the overall individual performance^4–6^, thanks to potential on-field transfer^7,8^. As a matter of fact, the development of specific tools that allow to evaluate or to improve visual-motor skills has found a favorable echo among psychologists, neurologists, neuroscientists, clinicians and sport professionals. Here, by capitalizing on flexibility of virtual reality technology, we aimed at implementing a visuomotor reaction task (Fig. 1), inspired by so-called Dynavision test^9^, in an attempt to identify major phenotypes associated with the spectrum of visual-motor response time performances.

The original visuomotor task requires a vertical board facing the subject, with buttons arranged in five concentric circles. The participant has to hit as quickly as possible buttons that successively light up and are switched off by said hit. The task places high demand on visual-perceptive and visual-cognitive functions and intends to evaluate eye-hand visuomotor abilities, with possible focus on specific visual search fields (upper, lower or peripheral)^1,9,10^. By providing a quantitative assessment of individual visuomotor integration, the task has demonstrated great value among healthy and diseased populations. Recently, normative values have been provided to help occupational therapy practitioners^11^. It is known that compromised ability to interactively coordinate visual perception and fine motor skills has been associated with a variety of neurological disorders and neurogenetic syndromes^12^. In elderly drivers who have suffered a stroke, psychomotor abilities such as visual information processing and allocation of attentional resources are degraded. Visuomotor reaction tasks have been fruitfully used to evaluate the degree of processing impairments, including individual peripheral awareness, a critical ability for safety driving^2^. More importantly, these very abilities have been improved thanks to a rehabilitation program based on using the Dynavision off-road task^13^. The task has also been successfully included in athletes’ visuomotor training programs^14^, even though the efficacy of retention and transfer to athletes’ performance in the field is a heated issue^7,15^. In this matter, including visuomotor reaction tasks in pre-season conditioning, then maintaining a vision training program during a season has been shown to improve batting statistics in college baseball players^4^.

Attentive processing in visual search has shown higher efficiency when the eyes and head directions are the same^16^. Alignment of the eyes and head prevents the division of attentional resources. Unfortunately, and despite obvious interest, the Dynavision task in its present form has limited capacities to address attentive visual strategies, because head movements are not quantified. Getting quantification of head movements is an asset that headsets for virtual reality can provide thanks to their included technology (gyroscopes, accelerometers). This technology may help going one step further in exploring individual abilities to process spatially distributed information, which offers interesting perspectives for assessing e.g. the recovery from brain damage. An interesting application could be the detailed diagnosis needed in sport experts who are exposed to concussions, which affect more than 3 million people each year in the United States^17^. As it stands nowadays, the diagnosis remains a challenge because it is quite exclusively based on the patient self-report. A visuomotor test can provide objective cues about peripheral vision response times that have been shown disproportionately prolonged after concussion^18^.

Remarkably, the original apparatus has been designed to contain an LCD display at (near) the center of the concentric circles of targets (see Fig. 1). Series of digit numbers can be successively presented throughout the visuomotor task, which requires sustained perceptual attention in central vision. Depending on the different modalities of the test (called ‘Standard’ and ‘Cognitive’ throughout the present work), subjects can be instructed to simply recite or more complicatedly manipulate digits, e.g. multiply or add them, which places higher cognitive demand. Logically when the cognitive demand increases, the number of successive hits in given time generally drops^10^. Since the creation of the apparatus^9^, the display capacity of digital technology have grown exponentially. Virtual reality has the capacity to place high and flexible demands on both visual-perceptive and visual-cognitive processing, which is an obvious asset to test eye-hand rapid coordination in neurologic rehabilitative programs, sport neurotraining or pilots conditioning, and to go one step further in exploring the complexity of visuomotor integration.

A number of factors have been documented that could shape individual visuomotor abilities. Video games practice is known to improve visual search in VR^19^. Action video games may specifically improve cognitive processes like spatial selective attention^20^ or skills such as problem solving, planning, visuomotor coordination or spatial cognition^21^. In combination with physical activity, perceptual-cognitive training has highlighted brain plasticity, which explains the emergence of particular behavioral phenotypes^14,22^. Among these factors, a sexual dimorphism has been noted and associated to sex-related implementation of spatial cognitive processes^23^ which matches with the consideration that gender is a confounding factor when relating cognitive flexibility to brain functional and effective connectivity^24^.

In the perspective of implementing a task in VR, one cannot exclude that VR per se imposes specific challenges that are absent in real world. Cybersickness has been described as being present in 25–50% of VR users^25^ and could be a confounding factor in visuomotor testing. Although the exact causes of cybersickness remain to be established, a conflict among multisensorial processing is pointed, where an incoherence between visual and vestibular inputs is a frequent candidate. VR may also induce a neurophysiological challenge to postural regulation when tasking upright in presence of incongruent inner ear and optical flow processing. Yet, all these phenomena represent a true concern when substantial optical flow is present in the virtual scene, which is not the case in the visuomotor VR task (VMVR) evoked here.

Here we present a VR-based evaluation of visuomotor response times that combined head movements recordings, and apply it among sport students. With the concern to consider multiple factors that intricate in visuomotor ability, we placed emphasis on a multifactorial approach.

## Methods

### Participants

Healthy sport sciences students (32 males, 32 females, 19.0 ± 2.1 years (range 17–25), 1.70 ± 0.09 m, 63.5 ± 9.6 kg, Body Mass Index 21.4 ± 2.3 kg/m^2^) gave their informed consent to participate to this program, that was part of their academic curriculum and for which they received credits. The institutional review board (Institutional Review Committee of The Faculty STAPS, Université de Bordeaux) approved the procedure that respected all ethical recommendations and followed the declaration of Helsinki. All the participants had normal or corrected-to-normal vision. None of them had any prior experience with the present task.

### Questionnaires

Before performing the visuomotor task in VR (VMVR) with different modalities, students had to fill a form in which they gave information about their sport practice in each of the last 3 years, as well as their video gaming habits. They also completed a Modified Edinburgh Handedness Questionnaire^26,27^. Immediately after the VMVR task, they filled another form to describe their own experience and feelings during the repeated VR tasks, including a translated version of the Simulator Sickness Questionnaire (SSQ, French version)^28^.

### Experimental setup

The experiment took place in a virtual environment (Fig. 1), developed by the CATIE (Centre Aquitain des Technologies de l'Information et Electroniques, Bordeaux, France) with the Unity software (Unity Technologies, San Francisco, USA), and delivered via an HTC Vive Pro headset with two manual controllers (HTC America, Inc. Seattle, WA, USA). The position of each controller in the VR environment were displayed as virtual gloves, thus constituting a visual feedback (Fig. 1). The scene consisted in an empty office including a replica of a the Dynavision apparatus (D2; Dynavision International LLC, West Chester, OH). Briefly, the virtual apparatus appears as a 120 cm × 120 cm board with 64 light-emitting buttons arranged in five concentric circles. The virtual board’s height was automatically adjusted in the visual field at the beginning of each experiment to fit the subjects’ height.

The visuomotor VR-based task (VMVR) was to hit as quickly as possible on the virtual vertical board the buttons that successively lit up and remained illuminated until touched by one of the HTC controllers held in the user’s hands. To assist the user in knowing that the target-button had been touched successfully, a sound was emitted through the headphones and a vibration occurred in the controller. Immediately afterwards, another button was illuminated elsewhere on the board in a random position.

### Procedure

The whole procedure in VR (including familiarization), lasted about 15 min during which the participant kept immerged with the headset in position; The procedure consisted in two similar one-minute runs repeated under four different modes: ‘*Standard*’ means that any button could light up; ‘*Cognitive*’ means that random 5-digit numbers are displayed every 5 s on the LCD screen (Fig. 1), and must be recited by the participants while concomitantly hitting buttons as described in ‘*Standard*’. ‘*Upper Visual Field (UVF)*’ and ‘*Lower Visual Field (UVF)* mean that only upper-half and lower-half buttons respectively can light up while participants were informed of that. The three most-distant buttons (upper and lower) were neutralized because not all participants could easily reach them^1^.

In agreement with previous recommendations for familiarization^10^, prior to executing the VMVR task in different modes, each participant ran two one-minute tests in *Standard* mode. During these pretests, participants were instructed to find a comfortable distance away from the virtual board so that they could touch and see all the buttons. In every condition, the height of the virtual board display was adjusted so that the participant’s eyes faced the LCD screen (see Fig. 1). Participants were instructed to preferentially switch-off the buttons on the left with the left controller, and conversely.

The subjects were randomly dispatched into eight groups to randomize the order of the modes (using the function alea() in an spreadsheet).

### Measurements from Unity software developments

During each run of the VMVR modes, the developed software allowed recording the number of successful hits, their position on the board, and the time between each strike. To prevent an excessive number of missed targets, a large hitbox around the virtual hand (controller) was tolerated. Sometimes, a participant hit a freshly illuminated button without realizing it, when repositioning its hand (controller) after a successful hit. This generated unrealistic short response times that were detected by considering < 200 ms after the previous successful hit and not taken into account in the further analysis. The average percentage of false detections for each subject, averaged across every condition, fell below 3% (2.1 ± 1.0%, range 0.3–4.2%).

Visuomotor Response Times (RTs) were obtained from the delay between the enlightening of a target button appearing in a random spatial position and the hit of said button with a handed VR controller. Therefore, RTs represent the combination of a visual reaction time (the time needed to identify the stimulus and initiate a reaction) and a motor execution time (the time needed to reach the target). RTs were averaged across 1-min, the duration of each run.

### Head movements (HMs) assessed by sensors in the VR headset

**Figure 2:**
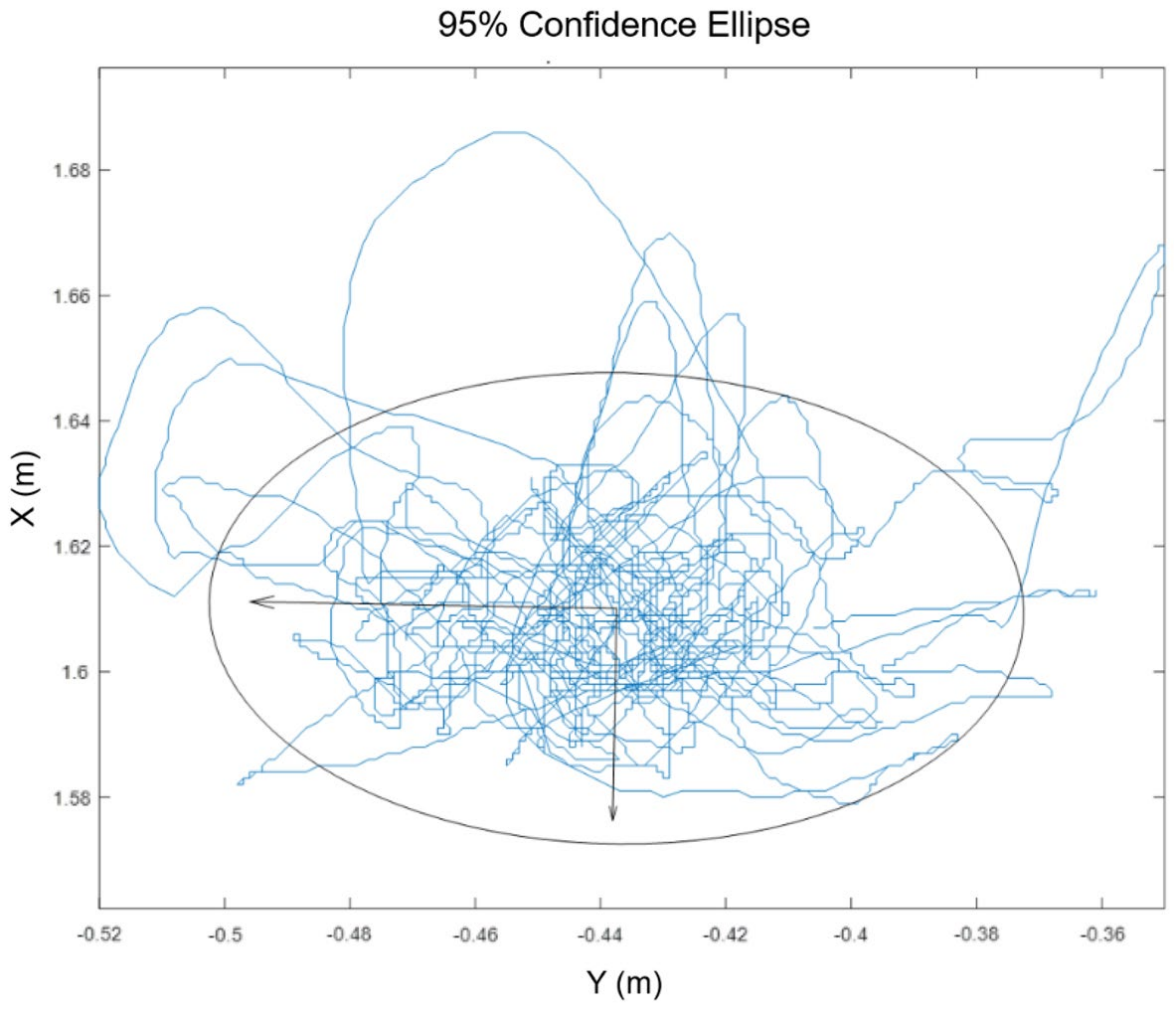
Example of a 95% confidence ellipse of head movements during VMRV in Standard condition.

Head movements were acquired from the HTC Vive headset using the SteamVR plugin (Fig. 2). The sampling frequency calculated from acquired data was 90.5 ± 5.5 Hz, similar to the Vive’s 90 Hz refresh rate. The data collected show a spatial resolution of 1 mm. As the tracking area was small and the subject stood at the same place, the HMD position error is below 10 mm, that has been accepted as suitable for experimentation and data exploitation^29,30^. We focused on head movements that were parallel to the plane of the virtual board, and exploited 2D-displacements along the x and y axes for this purpose. To quantify head movements, we computed the 95% confidence ellipse (Fig. 2) of the head’s trajectory. Ellipse computations were made in Matlab (Matlab 2019b, Matworks, Natick, MA, USA) according to previous recommendations^31^ from the projection of head movements in the 2D frontal plane.

### Statistics

Normal distribution for condition-dependent RT samples was assessed using a Shapiro–Wilk test. Response times (RTs) and head movements (HMs) were computed for each mode during test and retest and are expressed in mean ± SD. We also assessed an indicator of overall individual performances by mixing the RTs among all modes for each participant, a RT called *Mixed* (see Table 2).

### Test–retest reliability

Paired-samples *t*-test were used to compare mean RTs and HMs across test–retest in each condition (Standard, Cognitive, UVF, LVF and Mixed). Test–retest reliability was assessed using the Intraclass Correlation Coefficient (ICC), the Standard Error of Measurement (SEM) and Bland–Altman plots^32,33^. Whereas previous Dynavision studies used ICC version 3.1^9,34,35^, it seems that ICC version 2.1, considering the effects of trials as a random factor, may be a better choice^10^. Following previous indications and guidelines^36^, we chose a two-way random, single measures, absolute agreement model (ICC_2.1_), stating that less than 0.5 are indicative of poor reliability, values between 0.5 and 0.75 indicate moderate reliability, values between 0.75 and 0.9 indicate good reliability, and values greater than 0.90 indicate excellent reliability. ICC_2.1_ was calculated using Matlab, based on^37^.

Standard error to the mean (SEM) and SEM% (expressed as a percentage of the mean) were used to show how much measured scores are spread around a ‘true score’. SEM was calculated from ICC_2.1_ values as follows: $SEM=SD\sqrt{1-{ICC}_{2.1}}$^32^, with SD representing the standard deviation of the scores from all subjects for both trials. SEM% was obtained by dividing SEM by the mean RTs from all subjects for each condition.

To evaluate agreement between test and retest results, Bland–Altman plots were constructed for each condition by plotting the between-test difference against the mean difference. All of the statistical calculations were performed using Matlab and XLSTAT (Addinsoft, 2019, XLSTAT statistical and data analysis solution, Long Island, NY, USA).

### Factor analysis of mixed data

Factor analyses (Principal Component Analysis, PCA and Hierarchical Clustering on Principal Components, HCPC) were performed using the R software (*R Core Team (2020). R: A language and environment for statistical computing. R Foundation for Statistical Computing, Vienna, Austria*). To explore associations among participant’s features and analyze a large-scale behavior, a mixed PCA that included qualitative and quantitative variables was performed. Only eigenvalues greater than 1 (factor contains the same amount of information as a single variable) and that met the Kaiser’s rule (the most commonly used approach to selecting the number of components) were retained.

HCPC was performed after taking account for the main dimensions retained from PCA. HCPC delineates clusters of individuals with similar characteristics and prior analysis by PCA limits the statistical signal-to-noise ratio by denoising data and transforming categorical data in either continuous ones or balanced groups of variables. The hierarchical tree considered in the present study used the Ward’s criterion that consists to a series of agglomerative steps. Subjects were successively combined into clusters, which maximized both internal homogeneity (within-cluster variation) and external heterogeneity (between-cluster variation)^38^. This method is based on the objective function known as the within-group sum of squares or the error sum of squares. In Ward's method of HCPC, the emerging cluster is determined by the least increase of the sum of squared Euclidean distance, thereby reducing the loss of information that is inevitably associated with cluster fusion.

## Results

### Acceptability of the VR-based task

The VMVR task was associated to reasonably low scores of Simulator Sickness Questionnaire (SSQ) (6.8 ± 4.1, range 0–20), knowing that scores > 10 indicate significant symptoms, scores > 15 are worrying, and scores > 20 indicates a simulator problem^39^. In our population, only 7 out of our 64 participants scored 10 or above and none scored > 20. Subscale scores in the SSQ were 2.4 ± 1.8 (range 0–8) for *Nausea* and 4.3 ± 2.9 (range 0–14) for *Oculomotor*. There was no correlation between SSQ scores and averaged RTs (r = 0.065, *p* = 0.612). No distinction in SSQ scores were evidenced as well among subgroups of Team Sport, Tracking Ball, Video Games participants (Table 1). Gender had no effect either (females 7.2 ± 3.8, males 6.3 ± 4.5, *p* = 0.40).

**Table 1:** Mean SSQ scores or subscale scores as a function of qualitative variable.

Condition/score	Yes	No	*p*-value
Team sport/SSQ	6.3 ± 3.6	7.1 ± 4.5	0.41
Tracking ball/SSQ	6.3 ± 3.6	7.2 ± 4.7	0.40
Video games/SSQ	5.7 ± 4.1	7.6 ± 4.0	0.08
Video games/nausea	2.1 ± 2.0	2.7 ± 1.7	0.18
Video games/oculomotor	3.6 ± 2.8	4.9 ± 2.9	0.09

Values are mean ± SD.

### Visuomotor response times (RTs)

Shapiro–Wilk tests indicated normal distribution of the data in each condition (all p-values > 0.53). An ANOVA with repeated measurements showed different RTs across the four conditions (*F* = 267.1,* p* = 6 × 10^–78^). Post-hoc Bonferroni tests indicated mode specific RTs (all p-values < 0.002, Cognitive > Standard > UVF > LVF).

Similar RTs between test and retest (paired samples t-test) were evidenced (Table 2) in Standard (*p* = 0.439) and LVF mode (*p* = 0.350); a slight but significant improvement in performance (RTs decreased) during retest was observed in UVF (*p* = 0.017) and Cognitive (*p* = 0.033). As a consequence, the overall individual RT scores (Mixed) obtained when averaging one’s scores obtained among all modes was slightly better during retest (*p* = 0.010). This was expected since it has been advised to carry out 2–3 training sessions before definitive testing if one wants to withdraw short-term effects of habituation to the test^1, 10^.

**Table 2:** Test–retest values and reliability metrics for mean response times and head movement.

	UVF	LVF	Standard	Cognitive	Mixed
**Response times (RTs)**					
Test (ms)	610.8	573.3	779.2	1001.3	702.8
Retest (ms)	607.2*	564.1	784.6	974.0*	695.3*
ICC_2.1_	0.913	0.846	0.855	0.748	0.944
SEM (ms)	21.85	21.89	39.48	72.08	16.68
SEM %	3.59	3.85	5.05	7.30	2.39
**Head movements (HMs, 95% ellipse)**					
Test (mm^2^)	4333.4	3202.2	13,228.4	13,949.2	8678.3
Retest (mm^2^)	4195.3	3086.6	13,716.7	13,352.3	8587.7
ICC_2.1_	0.887	0.891	0.883	0.891	0.965
SEM (mm^2^)	956.3	856.4	2375.2	2288.2	747.8
SEM %	22.4	27.2	17.6	16.8	8.7

*p < 0.05 between test and retest.

### Head movements (HMs)

Shapiro–Wilk tests for head movements (95% confidence ellipse) showed the absence of normal distribution (all p-values < 0.01). Thus, a Kruskal–Wallis ANOVA test was used that demonstrated different HMs across conditions (Chi^2^ = 158.1, p = 5 × 10^–34^). Post Hoc Bonferroni tests indicated lower HMs in UVF and LFV when compared to HMs in Standard and Cognitive (all p-values < 0.001); yet, they were no difference between UVF and LVF (p = 0.3) and between Standard and Cognitive (p = 1.0). The profile for HMs was thus Standard = Cognitive > UVF = LVF.

### Response times (RTs) vs. head movements (HMs)

We showed the absence of correlation between RTs and HMs in each condition (all r < 0.2 and p > 0.10).

### Test–retest reliability among RTs and HMs

The level of test–retest reliability for RTs was assessed as good in Standard (ICC_2.1_ = 0.855) and in LVF (ICC_2.1_ = 0.846), excellent in UVF (ICC_2.1_ = 0.913), and moderate in Cognitive (ICC_2.1_ = 0.748). Taken together the whole RT performances demonstrated excellent test–retest reliability (Mixed, ICC 2.1 = 0.944).

In the same vein, HMs quantified by 95% confidence ellipses showed good test–retest reliability in every condition as demonstrated ICC_2.1_ coefficients > 0.88 (Table 2). This resulted in an excellent test–retest reliability (Table 2) when considering mode-mixed performances (Mixed, ICC_2.1_ = 0.965), which proved the robustness of HMs as a new cue to explore visual-perceptual strategies.

### Bland Altman plots

**Figure 3:**
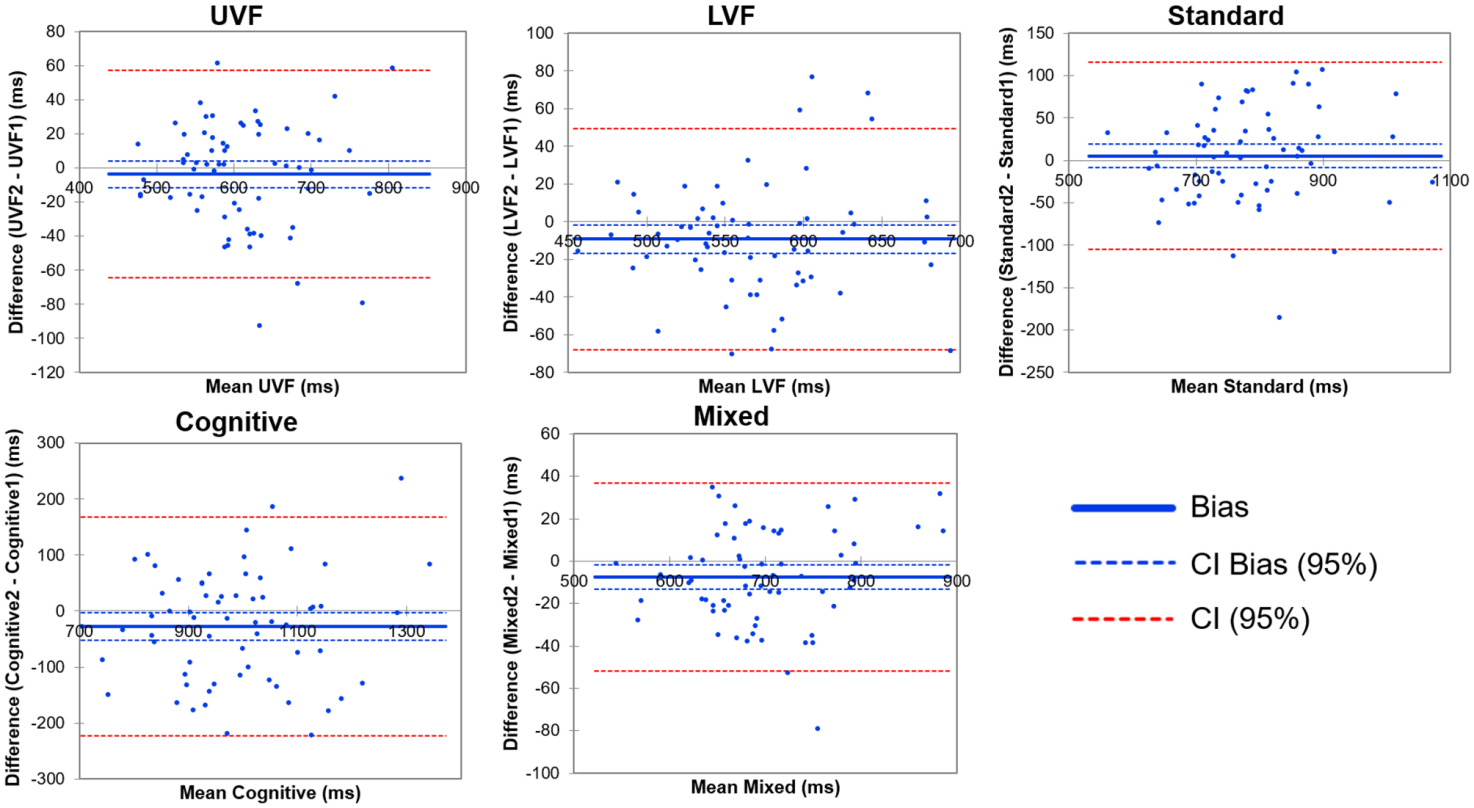
Bland Altman plots for test retest in each mode.

Bland Altman plots aim at showing outliers and bias. Here we show only a few outliers (outside the 95% confident interval) in each mode of the VMVR task. More importantly as regard bias, to evaluate the agreement between test and retest, the method looks at the average of the differences between the paired data, which is ideally zero and otherwise quantifies a bias. Figure 3 shows that the ideal value zero falls inside the 95% confidence interval of the mean bias value, which indicates good agreement between test and retest. A visual inspection of both bias and confidence limits shows that specifically Standard, and mixed data indicated good agreement.

### Principal component analysis of mixed data

**Figure 4:**
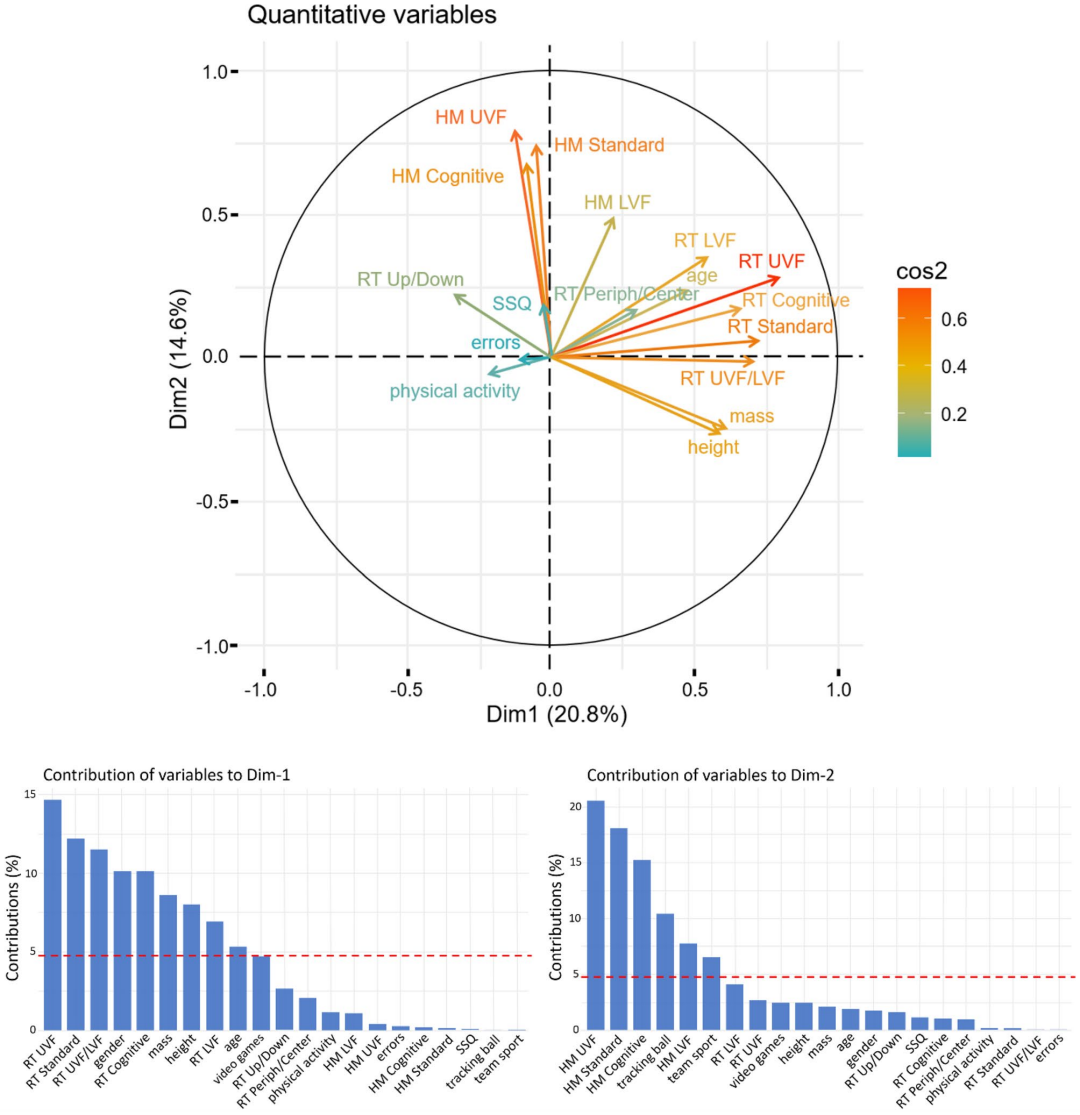
Upper panel: Principal Component Analysis of mixed data with the two main dimensions projected on orthogonal axes. RT performances in each mode actually represent mean test–retest 1/RT so that a high value indicates a great performance (quick response). HMs cues represent the mean test–retest 95% confidence ellipse of head movements in each mode. Physical Activity represents one’s weekly hours of practice. Errors indicates the number of one’s false verbally reported digits displayed during Cognitive condition. RT Periph/Center and RT Up/Down represent performances in the peripheral vs. (any of the) inner circles and upper- vs. lower-hemisphere hits recorded in Standard + Cognitive. Only quantitative variables are represented. Higher values for each variables are positively scattered along the components axis. Lower panels: contribution of each variable to the first two dimensions of the PCAmix illustrated by orthogonal axes. The dotted line corresponds to the expected value if the contribution of each variable was uniform. Contributions for qualitative variables represents ‘male’ over ‘female’ for gender, ‘yes’ over ‘no’ for video-game practice, and ‘no’ over ‘yes’ for team sports and tracking ball sports practice.

**Figure 5:**
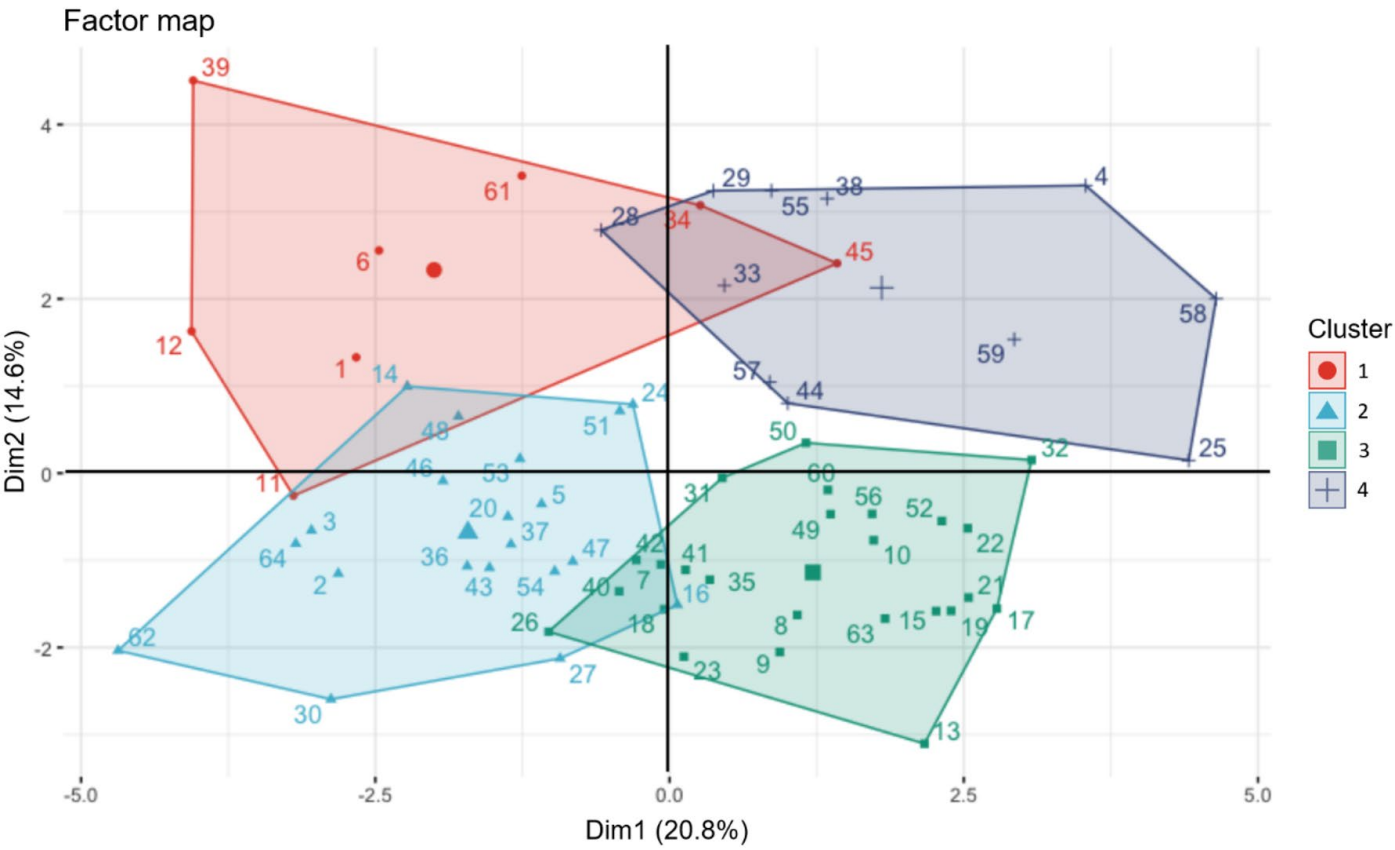
Mapping of the clusters as a function of PCAmix axes: Dim2 y-axis, Dim1 x-axis as in Fig. 4. Individuals are numbered and colored according to their cluster. The barycenter of each cluster is represented by a larger symbol.

To take into account the multiple factors intricating in individual visuomotor profiles, the whole set of variables (quantitative and qualitative) was submitted to a hierarchical clustering on principal component analysis which comprises two steps. First a principal component analysis was computed to disclose covariations among variables, to select the two main PCA dimensions and to unravel the main contributors for each dimension (Fig. 4). Then a hierarchical clustering on the two principal components was performed to delineate clusters of participants with similar characteristics (Table 3 and Fig. 5).

**Table 3:** Cluster description by the qualitative and quantitative variables.

Quantitative Var	v.test	*p*.value	Qualitative Var	v.test	*p*.value	Cla/Mod	Mod/Cla	Global
**Cluster 1**								
HM cognitive	5.0	0.000	None					
HM standard	4.8	0.000						
HM UVF	4.3	0.000						
RT standard	− 3.4	0.001						
RT up/down	3.2	0.001						
HM LVF	2.8	0.004						
RT cognitive	− 2.5	0.012						
RT UVF	− 2.4	0.015						
RT LVF	− 2.2	0.028						
**Cluster 2**								
Height	− 4.5	0.00001	Gender = F	5.7	1.15E−08	63	100	50
RT UVF/LVF	− 3.3	0.00113	Video games = No	3.1	1.81E−03	47	85	56
RT UVF	− 3.0	0.00239						
HM standard	− 3.0	0.00265						
Mass	− 3.0	0.00275						
Age	− 2.9	0.00349						
HM cognitive	− 2.8	0.00485						
HM LVF	− 2.7	0.00698						
RT standard	− 2.1	0.03857						
**Cluster 3**								
Height	5.0	0.0000005	Gender = H	6.1	8.56E−10	75	96	50
Mass	3.7	0.0002304	Video games = Yes	4.7	3.28E−06	71	80	44
HM UVF	− 3.2	0.0014187	Tracking ball = Yes	2.2	2.95E−02	51	72	55
RT UVF/LVF	2.6	0.0084472						
HM standard	− 2.2	0.0298603						
HM cognitive	− 2.1	0.0340746						
**Cluster 4**								
RT UVF	4.9	0.000001	Tracking ball = No	2.6	0.009992	31	82	45
RT LVF	4.8	0.000002	Team sport = No	2.0	0.042034	26	82	53
RT standard	3.8	0.000175						
age	3.6	0.000280						
RT cognitive	3.6	0.000318						
HM UVF	2.6	0.009153						

The column ‘v.test’ represents the statistical value used to determine the significance of the variables describing the group (a positive value indicates an over-representation of the modality under consideration; a negative value represents an under-representation). The column ‘Cla/Mod’ indicates what percentage of all individuals with this modality are in this cluster. For example, almost 63% of the females are in cluster 2. The column ‘Mod/Cla’ indicates what percentage of all individuals in the cluster have this modality. For example, 100% of subjects classified in Cluster 1 are females. The column ‘Global’ indicates the percentage of subjects out of the total population with this modality.

From PCA, we retained two main factors: dimension 1 (Dim1) and dimension 2 (Dim2), that explained respectively 21% and 15% of the total variance (Fig. 4). The most weighted contributions to Dim1 were response times (RT) performances, mainly in upper view field and Standard conditions. In order to show best performers clearly, we included 1/RTs instead of RTs; this way, a high value (quick response) indicates a great performance. Likewise, higher values for HMs show more head movements. Among other variables, *male (gender* = *1)*, *height* and *mass* contributed significantly to Dim1 (Fig. 4, lower panel). This indicates that globally tall males tended to be best performers and small females worst performers. The highest contributions to Dim2 in PCA were clearly head movements (HMs). The orthogonality between Dim1 and Dim2 in PCA indicates the absence of covariation in RTs and HMs. Of note, physical activity (in our physically-trained population) and cybersickness were very poor contributors to total variance.

### Hierarchical clustering on principal components (HCPC)

As a second step HCPC was performed, subsequently to PCA that is viewed as a denoising method which separates signal and noise where the two first dimensions extract the essential of the information. Without the noise in the data thanks to prior PCA processing, the clustering is more stable than the one that could be obtained from the original distances^38^. HCPC provided critical cues to highlight major visuomotor phenotypes, which is a critical outcome of the present study. Those cues are resumed in Table 3 and Fig. 5.

The HCPC returned a set of four clusters (Fig. 5 and Table 3), grouping participants that are broadly similar to each other. Each participant can be positioned within the cluster he belongs to, on a factor map to visualize individual positions in relation to Dim1 and Dim2 of the PCA. The individual position of participants in relation to Dim1 and Dim2 (orthogonal axes) indicate respective contributions of RTs (main contributor to Dim1) and HMs (main contributors to Dim2) in the phenotypes identified by HCPC clusters.

As shown by the position of cluster 1 in Fig. 5 and considering the contribution of variables to the dimensions of the PCA (Fig. 4), the most weighted variable for cluster 1 was HMs. Thus, this group of participants made extensive use of head movements (Dim2). As observed above in PCA orthogonality of Dim1 and Dim2, large head movements, a characteristic of this cluster of participants, cannot be qualified of an effective strategy since HMs do not covary with RT performances.

As indicated by metrics in Table 3, participants grouped by the HCPC in Cluster 2 were mainly females, small-sized, with great difficulties to score in so-called upper view field. Moreover, the majority of the participants in cluster 2 did not practice video-games (Table 3). The position of the cluster along Dim 1 in Fig. 5 indicates rather poor RT performances.

Cluster 3 was defined by participants who are tall (Table 3), reached great performances with limited head movements (Table 3 and Fig. 5), mainly in so-called upper field view (Table 3). Of note, > 70% of the people in cluster 3 were video-gamers and practice ball-tracking sports.

Best performers pertained to cluster 4 (Fig. 5). They scored in similar way in upper, lower and whole field of view modes (Table 3) while using large head movements (Table 3 and Fig. 5). They did not practice tracking-ball sports, nor team sports.

## Discussion

The main finding of the present study was that the visuomotor task implemented here in virtual reality (VMVR), inspired by the Dynavision visuomotor reaction task, not only provides reliable evaluations of classic visuomotor cues, but also provides new and reliable information on head movements. Cues on head movements are essential for exploring visuomotor phenotypes, as demonstrated here subsequently by the hierarchical clustering on principal component analysis. Remarkably, the new VR modality of the task brought about no drawbacks among our participants; they self-reported the absence of negative feelings about comfort and cybersickness and we show the absence of correlation between theses cues and response time performances (RTs). Considering the interest for such a visuomotor task in evaluating visuomotor plasticity, and the flexibility of VR technology, the task implemented here in VR is likely a promising tool for research in psychology, neuroscience, sport science and clinical practice.

As a first step in the present study, we evaluated test–retest reliability among main metrics. The computation of test–retest ICC_2.1_, SEM and SEM% demonstrated high consistency, in agreement with examination of the task reliability in real world that used the same metrics^10^. For Standard condition, previous studies found SEMs amounting to 21 ms^34^ and 43 ms^10^, which is in agreement with values obtained here in VR (SEM 39.5 ms, Table 2).

Yet, it should be stressed that we observed slightly higher response times in VR when compared to previous performances obtained in a quite similar population. Using standard conditions and a hard board, Wells et al.^10^ obtained averaged response times amounting to 755 ms and 710 ms in the Standard condition, which represents 3–10% better performances when compared to those obtained in our participants under VR conditions (Table 2). This does not call into question the validity of the VR task implemented here, and might have a putative origin in the motor component of the visuomotor response. Indeed, it has been shown that reaching a target takes longer time in VR when compared to non-VR conditions, which has been associated to a longer deceleration phase of the movement in the few instants before reaching the target^40^. As a whole, VR movements recruit more body segments than movements in real world and demonstrate a degree of behavioral uncertainty. Supposing that uncertainty rapidly vanishes with task repetition^40^, this likely explains slightly better performances observed here during retest. This may happen mostly among uncommon conditions (here Cognitive and UVF), with more marked initial uncertainty during test, but rapid adaptation and a marked RT progress during retest. Overall, these observations reinforce the recent suggestion that a number of trials are needed to become familiar with any visuomotor tasks in VR—a consign that is certainly more prevalent than in the real world—in order to obtain reliable assessments of visuomotor profiles.

Here we got a fine distinction of visuomotor phenotypes among sport students thanks to the VMVR task associated to the capture of head movements. Getting finer analyses of visuomotor integration abilities has several benefits such as screening neurological soft signs^41^ or tracking early progress in rehabilitation^13^ and vision training programs^14^. Here by adding head movement analysis, we discriminated individual behaviors in eye-hand coordination among young and healthy people, which is promising for exploring visuomotor phenotypes in more heterogeneous groups. First, we demonstrated that in our population, response time performances are not conditioned by head movements, as shown by orthogonality of both factors in PCA (Fig. 4). Although it has been shown that the alignment of eyes and head prevents the division in attentional resources during attentive processing in visual search^16^, such a behavior did not obviously represent an effective strategy in our conditions. In this vein, two distinct clusters embraced the best performers, that are separated by opposite head movement behaviors (clusters 3 and 4, Table 3). The ability of reaching high performance to the VMVR task while keeping the head more fixed (cluster 3) certainly relies on efficient peripheral vision, a key purpose in evaluating safety car driving^2,42^ or recovery after concussion in athletes^18^. Despite the absence of eye tracker in our experiments to confirm lateral viewing strategies, we noted that a number of factors covaried with the ‘head-fixed’ behavior. First, individuals characterized by fixed-head were 71% video-gamers, and correlatively 80% of the video-gamers in the whole population pertained to this cluster. This result is in line with previous demonstrations that tasks that rely on the dorsal system of the brain and peripheral vision are most improved in action video game players^43^ and that video gaming develops more efficient perceptual decisions^44^. Second, 70% of tracking-ball experts were in the fixed-head cluster and reciprocally 80% no tracking-ball were in the cluster represented by head moving performers (Fig. 5). Team sports and racquet sports require following the ball/object without losing information about the opponent(s) and the game area. These phenotypes support our interpretative hypothesis that VMVR combined with head movements analysis adds significant value for sport professional testing.

It is worth noting that those individual pertaining to the cluster defined by video-games and tracking ball also demonstrated greatest response time performances when upper view field is compared to lower view field processing. Although the origin of a specific phenotype associated with visual field processing remains to be identified (possibly the density of superior hemi-retina ganglion cells^1^, neural processing through the superior parieto-occipital cortex^45,46^ or a combination of both), the present analysis provides a coherent identification of individual abilities to process upper view field signals thanks to the analytic combination of response time performance, head movement and qualitative cues.

A sexual dimorphism has been reported in previous usage of a visuomotor task, which found no clear explanation^47^. Remarkably in our conditions cluster 2 embraced 100% females. In this cluster, the most weighted variables covarying with gender was height (negatively correlated) and poor RTs, mostly in upper view field. Therefore, it is hypothesized that height rather than sex per se, is a strong determinant of RTs in the VMVR task, especially when upper targets have to be reached. Likewise, height was positively correlated with RTs in clusters grouping best performers with fixed-head (Table 3, v.test). A striking consequence is that height may be a confounding factor in evaluating visuomotor ability when this ability assessed with an inadequate (virtual or real) board size. We observed that the smallest participants showed difficulties reaching the farthest buttons in our conditions, and came spontaneously closer to the virtual 1.2 × 1.2 m board, then diminishing their peripheral field of view. This could represent a methodological concern. Again, the visuomotor task in VR can bring significant improvements thanks to VR display flexibility. It is quite conceivable that the virtual board in VR has an adjustable size that takes body size (e.g. span) into account, which may bring more reliable quantification of pure visuomotor integration abilities.

While associating mental calculation to the visuomotor task through the display of digits on the board has undeniable interest to explore visual-cognitive functions, the lack of discriminatory power of the Cognitive mode among our participants calls for a comment. It may rely on a poor ratio cognitive load over cognitive status in our population. The population here was young, healthy and physically-trained spending many hours a week working out. In such people, reading and verbalizing numbers while coordinating eye-hand movements certainly represents very little difficulty in a dual-task setting. Rather, placing high mental demand relatively to the user’s cognitive capacity makes sense as demonstrated recently to distinguish common and distinct visuomotor impairments in autism and schizophrenia^41^. This is where VMVR has obvious advantages because of VR flexibility to design cognitive demands that can be adapted to the user (either children, elderly or athletes) as well to their impairment. Although it was not within the scope of the present study, designing specific cognitive loads in VMVR has an undeniable potential in clinical settings, neurodevelopmental research and conditioning athletes or pilots. The use of the VMVR task deserves further experiments in such specific populations. In addition, as VR technology is flexible and as VR set-ups are wearable and inexpensive nowadays, more participants could be engaged together in the same program, and/or home-practice could support rehabilitation or neurotraining programs.

In conclusion, extending practice and research in visuomotor domains will certainly be facilitated by the use of VR technology, as shown here with VMVR. Here we show good test–retest reliability, no manifestation of cybersickness and identifiable visuomotor phenotypes. The capture of head movements thanks to headset sensors offers clear additional advantages for clinical applications, psychophysiological research and sport training. Future works could assess whether body size is a confounding factor.

## Data Availability

The datasets generated and analyzed during the current study as well as the VMVR task are available from the corresponding author on reasonable request.

## References

[1] Stone SA (2018). Visual field advantage: Redefined by training?. Front. Psychol..

[2] Klavora P (1995). The effects of Dynavision rehabilitation on behind-the-wheel driving ability and selected psychomotor abilities of persons after stroke. Am. J. Occup. Ther. Off. Publ. Am. Occup. Ther. Assoc..

[3] Crotty M, George S (2009). Retraining visual processing skills to improve driving ability after stroke. Arch. Phys. Med. Rehabil..

[4] Clark JF, Ellis JK, Bench J, Khoury J, Graman P (2012). High-performance vision training improves batting statistics for University of Cincinnati baseball players. PLoS ONE.

[5] Feldhacker D (2019). Efficacy of high-performance vision training on improving the reaction time of collegiate softball athletes: A randomized trial. J. Sports Med. Allied Health Sci. Off. J. Ohio Athl. Train. Assoc..

[6] Schwab S, Memmert D (2012). The impact of a sports vision training program in youth field hockey players. J. Sports Sci. Med..

[7] Formenti D (2019). Perceptual vision training in non-sport-specific context: Effect on performance skills and cognition in young females. Sci. Rep..

[8] Burris K (2018). Sensorimotor abilities predict on-field performance in professional baseball. Sci. Rep..

[9] Klavora P, Gaskovski P, Forsyth R (1994). Test–retest reliability of the Dynavision apparatus. Percept. Mot. Skills.

[10] Wells AJ (2014). Reliability of the dynavision^T^ d2 for assessing reaction time performance. J. Sports Sci. Med..

[11] Blackwell, C. *et al.* Dynavision normative data for healthy adults: Reaction test program. *Am. J. Occup. Ther.* **74**, 7401185060p1–7401185060p6 (2020). 10.5014/ajot.2020.036251 PMC7018469 32078511

[12] Bueichekú E (2020). Central neurogenetic signatures of the visuomotor integration system. Proc. Natl. Acad. Sci. U. S. A..

[13] Klavora P, Warren M (1998). Rehabilitation of visuomotor skills in poststroke patients using the Dynavision apparatus. Percept. Mot. Skills.

[14] Ong NCH (2020). The use of Dynavision in sport and exercise research: A review. Int. J. Sport Exerc. Psychol..

[15] Broadbent DP, Causer J, Williams AM, Ford PR (2015). Perceptual-cognitive skill training and its transfer to expert performance in the field: Future research directions. Eur. J. Sport Sci..

[16] Nakashima R, Shioiri S (2014). Why do we move our head to look at an object in our peripheral region? Lateral viewing interferes with attentive search. PLoS ONE.

[17] Harmon KG (2013). American Medical Society for Sports Medicine position statement: Concussion in sport. Clin. J. Sport Med. Off. J. Can. Acad. Sport Med..

[18] Clark JF, Ellis JK, Burns TM, Childress JM, Divine JG (2017). Analysis of central and peripheral vision reaction times in patients with postconcussion visual dysfunction. Clin. J. Sport Med..

[19] Green CS, Bavelier D (2007). Action-video-game experience alters the spatial resolution of vision. Psychol. Sci..

[20] Green CS, Bavelier D (2003). Action video game modifies visual selective attention. Nature.

[21] Feng J, Spence I, Pratt J (2007). Playing an action video game reduces gender differences in spatial cognition. Psychol. Sci..

[22] Romeas T, Faubert J (2015). Soccer athletes are superior to non-athletes at perceiving soccer-specific and non-sport specific human biological motion. Front. Psychol..

[23] Waller D (2000). Individual differences in spatial learning from computer-simulated environments. J. Exp. Psychol. Appl..

[24] Chén OY (2019). Resting-state brain information flow predicts cognitive flexibility in humans. Sci. Rep..

[25] McIntire, J. P., Havig, P. R. & Geiselman, E. E. What is 3D good for? A review of human performance on stereoscopic 3D displays. In *Head- and Helmet-Mounted Displays XVII; and Display Technologies and Applications for Defense, Security, and Avionics VI* vol. 8383 83830X (International Society for Optics and Photonics, 2012).

[26] Oldfield RC (1971). The assessment and analysis of handedness: The Edinburgh inventory. Neuropsychologia.

[27] Cohen, M. S. Handedness Questionnaire. (2008).

[28] Bouchard S, St-Jacques J, Renaud P, Wiederhold BK (2009). Side effects of immersions in virtual reality for people suffering from anxiety disorders. J. CyberTher. Rehabil..

[29] Niehorster DC, Li L, Lappe M (2017). The accuracy and precision of position and orientation tracking in the HTC vive virtual reality system for scientific research. Percept..

[30] Luckett, E. A Quantitative evaluation of the HTC vive for virtual reality research. **31**.

[31] Duarte, M. Comments on “Ellipse area calculations and their applicability in posturography” (Schubert and Kirchner, vol.39, pages 518–522, 2014). *Gait Posture* **41**, 44–45 (2015). 10.1016/j.gaitpost.2014.08.008 25194690

[32] Weir JP (2005). Quantifying test–retest reliability using the intraclass correlation coefficient and the SEM. J. Strength Cond. Res..

[33] Kottner J (2011). Guidelines for reporting reliability and agreement studies (GRRAS) were proposed. Int. J. Nurs. Stud..

[34] Wells AJ (2013). Phosphatidylserine and caffeine attenuate postexercise mood disturbance and perception of fatigue in humans. Nutr. Res..

[35] Klavora P, Gaskovski P, Forsyth RD (1995). Test–retest reliability of three Dynavision tasks. Percept. Mot. Skills.

[36] Koo TK, Li MY (2016). A guideline of selecting and reporting intraclass correlation coefficients for reliability research. J. Chiropr. Med..

[37] Salarian, A. Intraclass Correlation Coefficient (ICC). https://www.mathworks.com/matlabcentral/fileexchange/22099-intraclass-correlation-coefficient-icc (2020).

[38] A review of clustering methods. In *Cluster Analysis* 34–62 (SAGE Publications, Inc., 1984). 10.4135/9781412983648.n3.

[39] Kennedy RS, Lane NE, Berbaum KS, Lilienthal MG (1993). Simulator sickness questionnaire: An enhanced method for quantifying simulator sickness. Int. J. Aviat. Psychol..

[40] Just M, Stirling D, Ros M, Naghdy F, Stapley P (2016). A comparison of upper limb movement profiles when reaching to virtual and real targets using the Oculus Rift: Implications for virtual-reality enhanced stroke rehabilitation. J. Pain Manage..

[41] Carment L (2020). Common vs distinct visuomotor control deficits in autism spectrum disorder and schizophrenia. Autism Res. Off. J. Int. Soc. Autism Res..

[42] Klavora P, Heslegrave RJ, Young M (2000). Driving skills in elderly persons with stroke: Comparison of two new assessment options. Arch. Phys. Med. Rehabil..

[43] Chopin A, Bediou B, Bavelier D (2019). Altering perception: The case of action video gaming. Curr. Opin. Psychol..

[44] Pavan A (2019). Visual short-term memory for coherent motion in video game players: Evidence from a memory-masking paradigm. Sci. Rep..

[45] Rossit, S., McAdam, T., McLean, D. A., Goodale, M. A. & Culham, J. C. fMRI reveals a lower visual field preference for hand actions in human superior parieto-occipital cortex (SPOC) and precuneus. *Cortex J. Devoted Study Nerv. Syst. Behav.* **49**, 2525–2541 (2013). 10.1016/j.cortex.2012.12.014 23453790

[46] Rossit S, Fraser JA, Teasell R, Malhotra PA, Goodale MA (2011). Impaired delayed but preserved immediate grasping in a neglect patient with parieto-occipital lesions. Neuropsychologia.

[47] Klavora P, Esposito JG (2002). Sex differences in performance on three novel continuous response tasks. Percept. Mot. Skills.

